# Vaccination of koalas during antibiotic treatment for *Chlamydia*-induced cystitis induces an improved antibody response to *Chlamydia pecorum*

**DOI:** 10.1038/s41598-020-67208-x

**Published:** 2020-06-23

**Authors:** Samuel Phillips, Bonnie L Quigley, Olusola Olagoke, Rosemary Booth, Michael Pyne, Peter Timms

**Affiliations:** 1 Genecology Research Centre, School of Science and Engineering, The University of the Sunshine Coast, Queensland, Australia; 2 Australia Zoo Wildlife hospital, Steve Irwin Way, Beerwah, Australia; 3 Currumbin Wildlife Hospital, Currumbin, Australia

**Keywords:** Immunology, Microbiology, Molecular biology

## Abstract

*Chlamydia* infection and disease are endemic in free-ranging koalas. Antibiotics remain the front line treatment for *Chlamydia* in koalas, despite their rates of treatment failure and adverse gut dysbiosis outcomes. A *Chlamydia* vaccine for koalas has shown promise for replacing antibiotic treatment in mild ocular *Chlamydia* disease. In more severe disease presentations that require antibiotic intervention, the effect of vaccinating during antibiotic use is not currently known. This study investigated whether a productive immune response could be induced by vaccinating koalas during antibiotic treatment for *Chlamydia*-induced cystitis. Plasma IgG antibody levels against the *C. pecorum* major outer membrane protein (MOMP) dropped during antibiotic treatment in both vaccinated and unvaccinated koalas. Post-treatment, IgG levels recovered. The IgG antibodies from naturally-infected, vaccinated koalas recognised a greater proportion of the MOMP protein compared to their naturally-infected, unvaccinated counterparts. Furthermore, peripheral blood mononuclear cell gene expression revealed an up-regulation in genes related to neutrophil degranulation in vaccinated koalas during the first month post-vaccination. These findings show that vaccination of koalas while they are being treated with antibiotics for cystitis can result in the generation of a productive immune response, in the form of increased and expanded IgG production and host response through neutrophil degranulation.

## Introduction

*Chlamydia* is a Gram-negative, obligate intracellular bacterium that is a pathogen in humans, domestic animals, livestock and wildlife^1^. *Chlamydia* species can infect a wide range of mucosal surfaces and present as symptomatic or asymptomatic infections^2^. In most hosts, conjunctival infections lead to inflammation of the conjunctival tissue and, in chronic infections, can result in ocular scarring and eventual blindness^3^. Infections of the reproductive mucosa can result in ascending infection of the female and male reproductive tracts and, in females, chronic infections can lead to the development of pelvic inflammatory disease and ovarian cysts, resulting in infertility^4–6^. Finally, infections of the uroepithelium lead to inflammation of the urethra and, in severe cases, inflammation of the bladder wall (cystitis), with chronic infections resulting in ascending ureter infections and eventual nephritis^7,8^. Further to these more common mucosal surfaces, recent evidence suggest that *Chlamydia* can infect the gastrointestinal tract, with both asymptomatic^9–13^ and symptomatic^14–16^ outcomes.

The Australian marsupial, *Phascolarctos cinereus* (koala), is listed as a vulnerable and protected species^17^. The significant decline of koala populations has been attributed to several anthropogenic factors as well as disease related to *Chlamydia pecorum* infections^7,18^. The koala is known as a specialist folivore, which has resulted in specific adaptations to both the gastrointestinal microbiome and physiology in response to its exclusive diet of eucalyptus leaves^19^. These adaptations complicate antibiotic treatment of koalas, resulting in the need for extended, high dose treatment periods, commonly leading to gastrointestinal dysbiosis^7,20–22^. Fortunately, a significant amount of research has been focused on the development of a *Chlamydia* vaccine in many different hosts, including koalas^1^. Significant efforts have shown the *Chlamydia* major outer membrane protein (MOMP) could be an ideal target for future vaccine development^1^.

A *Chlamydia* vaccine for koalas has been under development for several years. The most tested version of the *Chlamydia* koala vaccine has demonstrated induction of humoral immune responses^23–27^ and, importantly, had a therapeutic effect (replacing antibiotic treatment) in koalas with mild conjunctival disease^23^. These studies used recombinant proteins representing three sequence types of the *C. pecorum* MOMP protein, combined with a three-component adjuvant. Although the results from this recombinant vaccine are promising, large scale production of recombinant protein is difficult and expensive^28^. Consequently, identification of two specific immunogenic regions of the MOMP has resulted in an updated, synthetic peptide-based version of the vaccine for koalas^2^. Nyari and colleges used two specifically designed peptides from MOMP to induce *C. pecorum* MOMP specific IgG and IgA antibodies able to recognise multiple MOMP genotypes and at levels similar to the recombinant MOMP vaccine^2^. It is believed that expansion of these synthetic peptides will induce an even greater response than observed in the previous trial.

A further challenge to vaccinating koalas is that the majority of koalas seen at wildlife hospitals arrive with clinical signs of disease, meaning that they require antibiotic treatment. So, unlike the mild conjunctival disease situation where vaccination could replace antibiotic treatment, many disease presentations, like cystitis in females, require antibiotic intervention on animal welfare grounds. However, given that the previous trial showed that a *Chlamydia* vaccine could have a therapeutic effect on ocular disease alone, this raised the question of whether vaccination in conjunction with antibiotic use could produce a greater positive effect on more serious disease presentations. The use of antibiotics, such as doxycycline and clarithromycin, have been demonstrated in mice to suppress the antibody responses to T-cell-dependent and T-cell-independent antigens during vaccination against hepatitis B virus and *Salmonella typhi*^29^. Therefore, the goal of this study was to evaluate whether a productive immune response could be induced by vaccinating koalas during antibiotic treatment for *C. pecorum*-induced cystitis.

The current study found that antibiotics have a short term negative effect on the presence of systemic anti-MOMP IgG antibodies in naturally diseased animals. However, koalas that were treated with antibiotics but also vaccinated, generated an improved IgG systemic immune response to the MOMP of *C. pecorum* in the weeks after antibiotic treatment had finished. Cellular expression analysis also detected the presence of an active cellular immune response with significant neutrophil degranulation pathways active in vaccinated koalas, during the first month post-vaccination. Finally, this study found that specific amino acid sequences within MOMP were recognised post-vaccination by way of increased IgG production and therefore these targets could be useful for future development of a peptide vaccine.

## Results

### Naturally infected and diseased koalas present with different systemic anti-MOMP IgG antibody profiles

**Figure 1:**
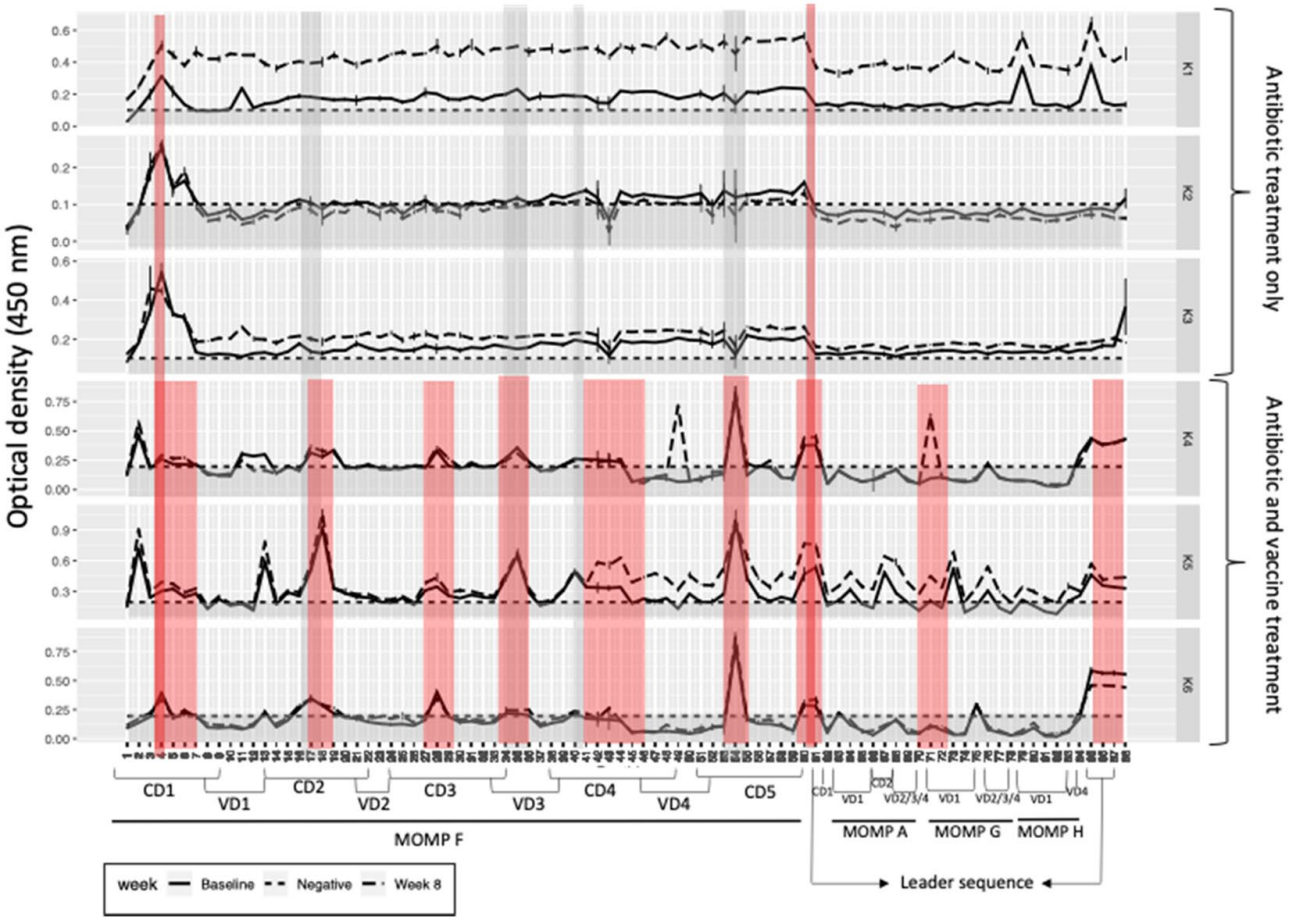
Systemic anti-MOMP IgG antibody responses in six koalas with natural infections and disease, measured at admission to AZWH and four weeks after antibiotic course completion. Vertical grey bars indicate epitopes recognised in ≥4 koalas (horizontal grey bar indicates negative control plus two standard deviations) full-length vertical red bars indicate epitopes recognised in vaccinated and un-vaccinated koalas and half-length vertical red bars indicate epitopes recognised in all vaccinated koalas), koalas K1, K2 and K3 are unvaccinated and koalas K4, K5 and K6 are vaccinated.

Epitope mapping was used to identify which regions of MOMP were recognised by plasma IgG antibodies from the six koalas which completed the trial. Interestingly, while each koala with *Chlamydia*-induced cystitis had a unique IgG antibody recognition pattern to the *C. pecorum* MOMP, there were six regions that elicited a response in four or more koalas. These regions were conserved domain (CD) one (epitope 4), two (epitopes 17 and 18) and five (epitopes 54 and 60) and the variable domain (VD) and CD overlap for CD3/VD3 (epitopes 34 and 35), VD3/CD4 (epitope 40) and VD4/CD5 (epitope 53) (vertical grey bars in Fig. 1).

### Negative effect of antibiotics on systemic anti-MOMP IgG antibodies in koalas

**Figure 2:**
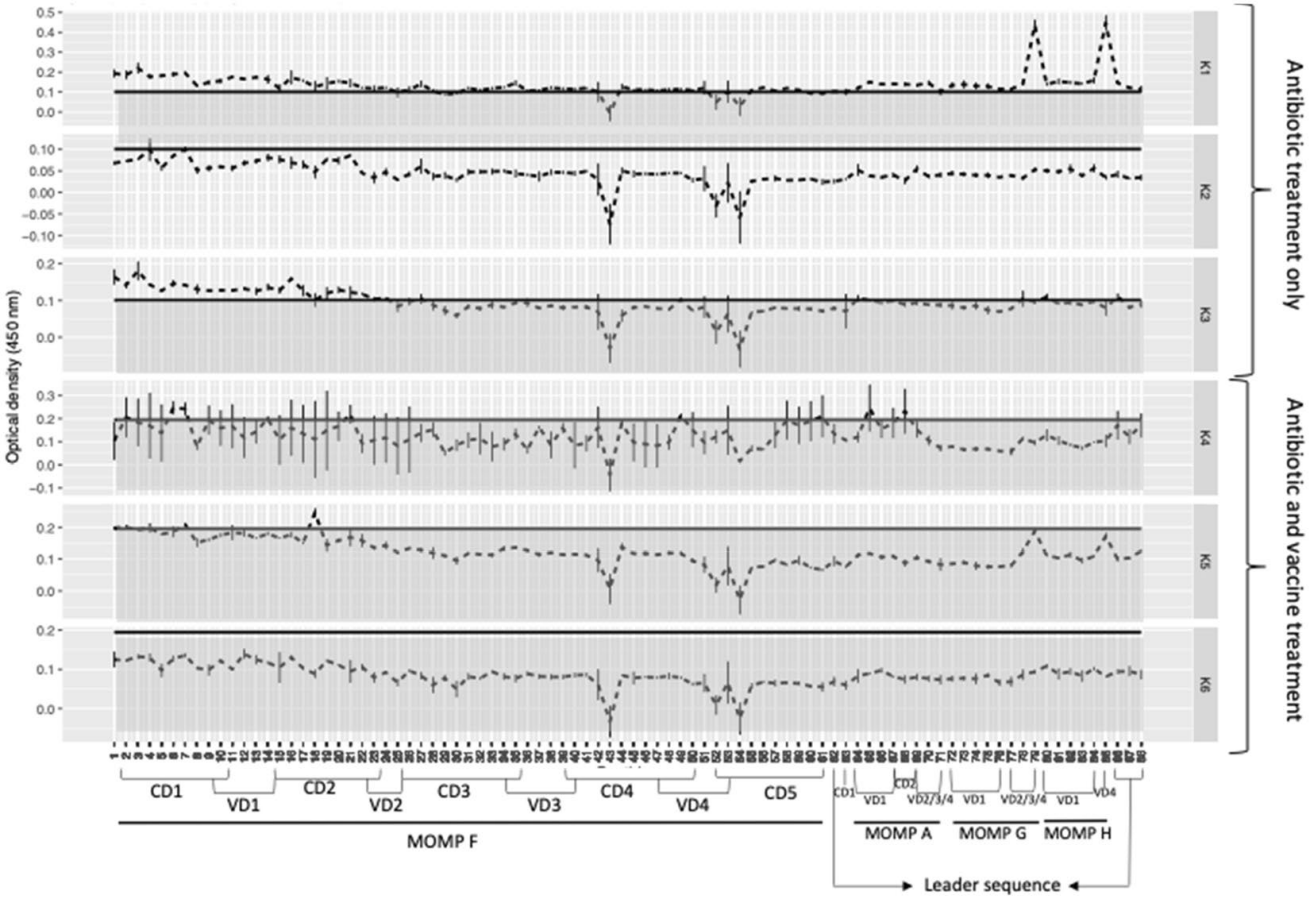
Systemic anti-MOMP IgG antibody responses in koalas treated with antibiotics for four weeks. Koalas K1, K2 and K3 are unvaccinated and koalas K4, K5 and K6 are vaccinated (horizontal grey bar indicates negative control plus two standard deviations).

After four weeks of antibiotic treatment, all six koalas had a decrease in IgG antibody recognition of MOMP (Fig. 2). In the unvaccinated group (K1, K2 and K3), only one out of three koalas (K1) retained a strong recognition of two epitopes (79 and 85) (Fig. 2). In the vaccinated koalas (K4, K5 and K6) two out of three animals retained some level of MOMP recoginition during the four week antibiotic period (Fig. 2).

### Vaccination improves systemic anti-MOMP IgG antibody response in koalas

At eight weeks into the trial, koalas were considered healthy and ready for release back into the wild. At this 8 week time-point, all three unvaccinated koalas showed recognition of CD one (epitope 4), two out of three (K1 and K2) showed recognition of CD five (epitope 60) and K1 still showed recognition of epitopes 79 and 85. By comparison, the three vaccinated koalas showed the presence of antibodies that recognised CD one (epitopes 4, 5, 6 and 7), two (epitopes 17, 18 and 19), three (epitopes 27, 28 and 29), four (epitopes 40, 41, 42, 43 and 44) and five (epitopes 53, 54, 60 and 61) and VD three from MOMP F (epitopes 34 and 35), one from MOMP G (epitopes 75 and 76) and the leader sequence (epitopes 86, 87 and 88) (Fig. 1).

### Antibiotics suppress humoral immune responses as compared to before and 4 weeks post antibiotic treatment

**Figure 3:**
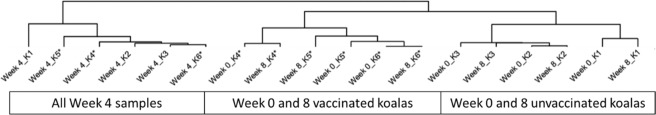
Similarity tree constructed using person’s correlation for all six koalas at weeks 0, 4 and 8 (samples from vaccinated koalas (K4, K5 and K6) are denoted as *).

Antibiotics, as anticipated, had an effect on antibody production to MOMP. A similarity analysis of the epitope mapped regions of the *C. pecorum* MOMP using Pearson’s correlation identified three distinct clusters with similar IgG recognition patterns; Samples from admission and week eight in vaccinated koalas, samples from admission and week eight and unvaccinated koalas and samples from all koalas at week four (Fig. 3). The combined week four cluster concurs with previous analysis that identified antibiotic treatment as having a negative effect on systemic IgG antibodies, regardless of vaccination status. Fortunately, this negative effect is only temporary, with all koalas regaining some of their naturally-induced systemic IgG antibodies after the completion of the course of antibiotics (Fig. 3). Furthermore, clustering identified that the antibody profiles of koalas at admission and eight weeks post-vaccination were more similar to each other than unvaccinated koalas at the same time points (Fig. 3). These similarities in antibody patterns pre-and post-vaccination suggest that vaccination enhanced the natural antibody production already present in a koala and confirms the trends observed from the biologically relevant groupings.

### Enhanced IgG antibody response to the MOMP conserved and variable domains in vaccinated koalas

**Figure 4:**
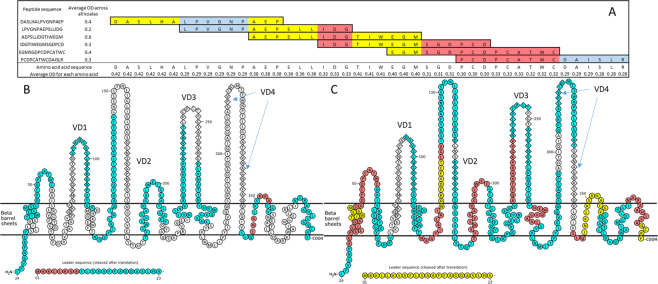
Predicted two dimensional structure of *C. pecorum* genotype F (DbDeUG strain) major outer membrane protein (MOMP) with amino acid scoring method. Diamonds represent variable domains, circles represent conserved domains. Coloured amino acids are regions recognised by MOMP IgG antibodies, where blue, red and yellow coloured amino acids had an average of combined optical density score equal to or greater than 0.2, 0.3 and 0.4, respectively. (**A**) Amino acid scoring method example. (**B**) Six koalas at admission. (**C**) Three koalas eight weeks post vaccination.

To focus in on which amino acids were linked to the strongest responses, the signal detected from each epitope mapping peptide was assigned to each amino acid in the peptide. Where peptides overlapped, the signal detected for each amino acid was averaged for the overlapping peptides (Fig. 4A). Using this assignment, the antibody binding pattern showed that during natural infection, IgG antibodies are made recognising sections of all five conserved domains, variable domain one and the first seven amino acids of variable domain three of MOMP F (Fig. 4B). Conversely, the three vaccinated koalas displayed enhanced IgG antibody binding with a greater intensity (up to twice the intensity in some regions) and an increase in recognised regions (Fig. 4C). This included recognition of amino acids from all five conserved and four variable domains (Fig. 4C). These results indicate that vaccination expanded the IgG antibody recognition of the MOMP beyond that induced during natural infection, with both naturally recognised amino acid sequences and new, previously unrecognised, amino acid sequences identified eight weeks post-vaccination. A comparison of the amino acid sequences recognised during natural infection and due to vaccination indicates nine new IgG recognised regions not previously identified in naturally infected koalas (Fig. 4C). Sections from all five conserved domains can be attributed to a vaccine response, with some regions of both variable domain two and four also only recognised post vaccination (Fig. 4C).

### Low levels of detectable anti-*Chlamydia* antibodies at mucosal sites highlight challenges in monitoring mucosal immunity

Mucosal swab samples from the conjunctiva, rectal and urogenital tracts were used to measure the IgG and IgA antibody recognition of both recombinant MOMP and heat inactivated *C. pecorum* genotype G elementary bodies (EBs). Unfortunately, the measured optical densities for the majority of samples were so close to the assay’s limit of detection, that meaningful analysis could not be undertaken. This combination of technical sensitivity limit, combined with the low levels of antibodies produced at these sites highlights the difficulties in measuring mucosal immune responses from living animals.

### Identification of 423 differentially expressed genes post-vaccination in koalas with current signs of *Chlamydia*-induced cystitis

Gene expression within circulating peripheral blood mononuclear cells (PBMCs) was assessed using RNA sequencing analysis from four unvaccinated koalas (two male and two female) and five vaccinated koalas (three male and two female) at 0, 2, 4, 6 and 8 weeks. Of the 39 data points available for analysis from the nine koalas, five samples (two unvaccinated male samples week 4 and 6, two vaccinated male samples week 0 and 2 and one vaccinated female sample week 0) failed quality control (multidimensional scaling plot outliers) and were subsequently removed from further analysis (Supplementary Figure 1).

Total RNA sequencing resulted in an average of 30.5 million reads per sample, with an average 9.2 million sequence reads per sample mapped to the koala reference genome. After quality control using a minimum of 10 counts per million in greater than three samples, a total of 12,401 genes were analysed using a quasi-likelihood generalised linear model approach to determine significantly expressed genes (Supplementary Figure 1). Two separate differential gene expression analyses were performed. The first comparison looked at the average gene expression between vaccinated and unvaccinated koalas during antibiotic treatment (weeks 0, 2 and 4) while the second comparison looked at vaccinated and unvaccinated koalas after antibiotic treatment (weeks 4, 6 and 8). This identified 228 differentially expressed genes (with an adjusted p value of <0.05) during antibiotic treatment and 195 genes (with an adjusted p value of <0.2) after antibiotic treatment (Supplementary Figure 1).

### Genome-wide overview of differentially expressed genes induced by vaccination

A genome-wide pathway analysis for all differentially expressed genes (DEGs) during and after antibiotic treatment, identified that the overall response to vaccination changed between the time frames assessed. During the first month post-vaccination (during antibiotic treatment), 14 different biological pathways were identified as being regulated (Table 1). In the second month post-vaccination (after antibiotic treatment), there were a total of 11 different biological pathways regulated (Table 1). Common biological pathways over both time frames included signal transduction, developmental biology, cell cycle, vesicle-mediated transport, metabolism, disease and immune system. Pathways unique to the first month post-vaccination included transport of small molecules, programmed cell death, cellular responses to external stimuli, gene expression and haemostasis. Pathways unique to the second month post-vaccination included metabolism of proteins, metabolism of RNA and neuronal system.

**Table 1:** Genome-wide pathway analysis of all genes identified as differentially expressed using the pathway analysis tool “Reactome”.

Genome-wide pathways	Pathways identified in the first month post-vaccination (during antibiotic treatment)	Pathways identified in the second month post-vaccination (after antibiotic treatment)
Immune System	9	2
Cell Cycle	3	3
Cell-Cell communication	1	1
Developmental biology	6	3
Disease	4	5
Metabolism	3	2
Signal Transduction	22	3
Vesicle-mediated transport	1	2
Transport of small molecules	2	0
Programmed Cell Death	3	0
Cellular responses to external stimuli	2	0
Gene expression	5	0
Haemostasis	3	0
Metabolism of proteins	0	3
Metabolism of RNA	0	3
Neuronal system	0	2

Each of these identified genome-wide processes contained biological pathways involving differentially expressed genes. During antibiotic treatment, the two pathways identified as significant (FDR values <0.001) were neutrophil degranulation and RHO GTPases activate citron kinase. After antibiotic treatment, only one significant pathway (FDR value of 0.017) was identified as UCH proteinases. Due to the immunological links to neutrophil degranulation, this report provides further analysis on the regulation of the specific genes in this pathway.

### The innate immune system and neutrophil degranulation is significantly regulated in the first month post-vaccination

**Figure 5:**
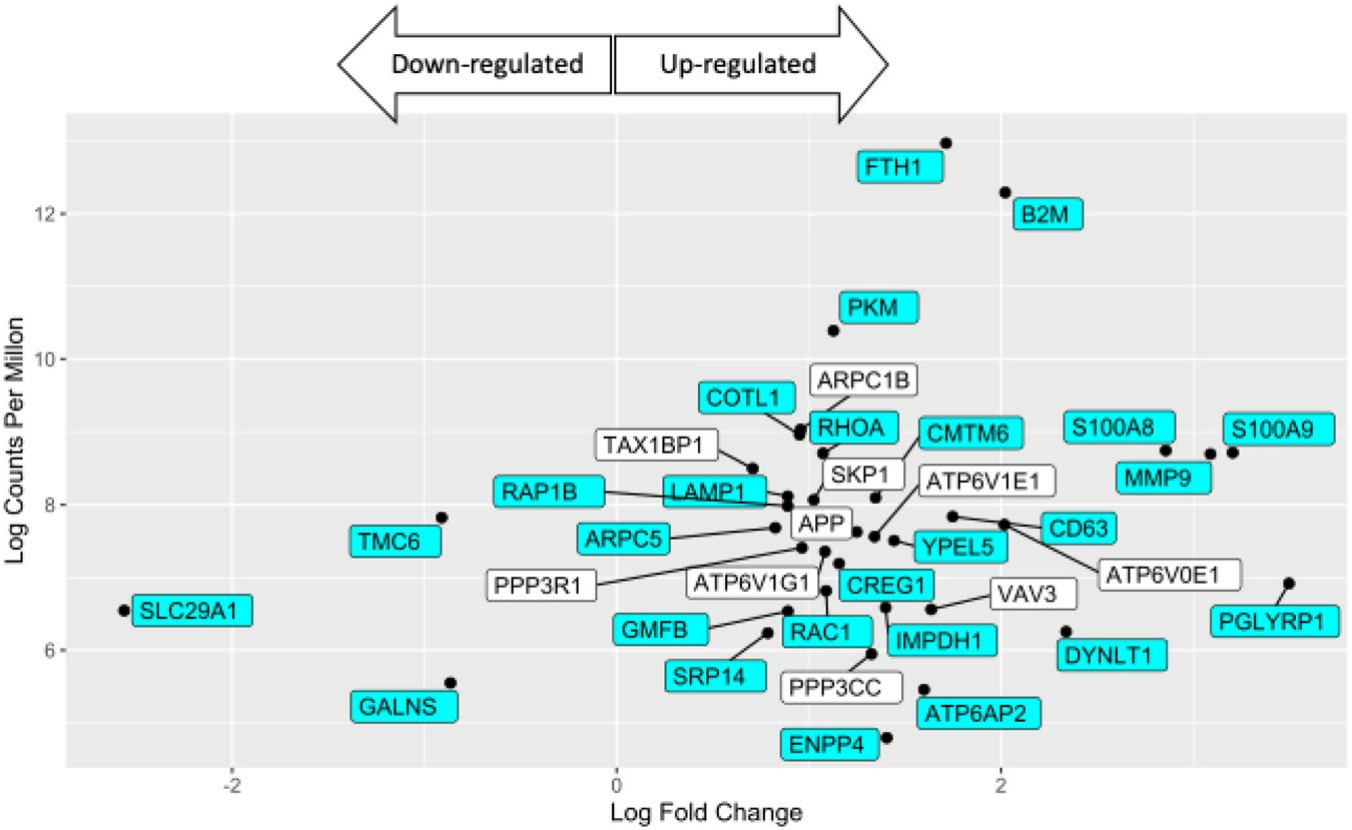
Expression of the 39 differentially expressed genes involved in the innate immune response detected in vaccinated koalas during the first month post-vaccination. Genes involved in neutrophil degranulation (29 genes) are coloured aqua. White genes are involoved in other innate immune responses.

Analysis of the 228 differentially expressed genes in the first four weeks post-vaccination indicated that 39 of these genes were involved in innate immune responses, with 29 of these genes being involved in neutrophil degranulation (FDR 6.54e^−07^) (Fig. 5, aqua coloured genes). Of the 480 genes known to be involved in neutrophil degranulation in some way, three genes (TMC6, GALNS and SLC29A1) were down-regulated in vaccinated koalas and 26 genes were up-regulated (Fig. 5).

## Discussion

In the current study, the effect of concurrently vaccinating koalas with signs of *Chlamydia*-induced cystitis while they were being treated with antibiotics was assessed. Due to the severity of urogenital *Chlamydia* infections in koalas, the use of antibiotics is necessary to limit disease progression. With previous trials indicating a therapeutic effect in ocular disease, it was hypothesised that vaccinating koalas while on antibiotics would enhance both the therapeutic and protective effects of the vaccine.

Immunomodulatory effects of antibiotics have been reported in many different trials and have demonstrated both positive and negative effects on both cellular and humoral immune responses^30^. In the current koala trial at admission (week 0), each koala was determined to have a unique *Chlamydia*-specific, plasma IgG antibody profile to MOMP by both the epitopes recognised and the intensity observed. This difference in profiles was not unexpected and is believed to be driven by host genetics, infecting *Chlamydia* genotype and the history of infections each koala has experienced during their lifetime.

Interestingly, at the end of antibiotic treatment (week 4) all koalas were observed to have a lowered IgG profile compared to the profile present at admission (week 0), regardless of vaccination status. This suppression of IgG antibodies has been demonstrated previously to a range of different antibiotics, including doxycycline treatment in both pig and mice trials^29,31^. Pomorska-Mol *et al*. (2016), demonstrated that pigs vaccinated with an inactivated vaccine against erysipelas and treated with either ceftiofur, doxycycline or tiamulin, resulted in a decrease of antigen specific circulating IgG antibody recognition. However, the opposite effect was observed with amoxicillin and tulathromycin, with enhanced effects on circulating IgG antibodies^31^. Woo *et al*. (1999) also observed differences in results depending on the antibiotic used, but also demonstrated opposing effects in the use of both clarithromycin and doxycycline, depending on the vaccine antigen. Antibody responses to tetanus toxoid, pneumococcal polysaccharide vaccine, and hepatitis B virus surface antigen vaccine were supressed. Conversely, antibody responses to live attenuated *Salmonella typhi* vaccine were enhanced during treatment with clarithromycin or doxycycline^29^. With the proven antibody suppression of doxycycline it is therefore expected that the same effect would be seen in koalas also treated with doxycycline.

Fortunately, once antibiotic treatment had finished, a return of IgG antibodies was observed in all koalas. In unvaccinated koalas, the circulating IgG antibody level and range observed post-treatment was equal to the level and range observed at admission. There was an observed increase in two unvaccinated koalas (K1 and K3) across the entire length of MOMP F, although this observation is thought to be the result of non-specific binding to the ELISA plate, as an increase in MOMP recognition after infection clearance does not make biological sense. In vaccinated koalas, both the circulating IgG antibody level and range, increased. This increase in epitope recognition post-vaccination has also been observed in *Chlamydia-*free koalas and koalas with current asymptomatic *Chlamydia* infections^24,26^. While these previous vaccine studies measured antibody responses at greater than 20 weeks post-vaccination, this is the first to report on this vaccine response within the first eight weeks post vaccination, indicating that vaccinated koalas have an increased anti-*Chlamydia* immunity within eight weeks post vaccination and aids planning for future vaccine schedule programs.

The recovery of an enhanced antibody response to the MOMP in koalas indicates that the antibody suppression due to antibiotics is only temporary. The lasting effect that antibiotics have on the humoral immune response has been demonstrated to depend on the antibiotic used, the dose schedule and the antigen used to vaccinate^30^. Bowden *et al*. (1998) identified that the time between vaccination and commencement of antibiotics can significantly affect the development of antigen specific IgG antibodies^32^. They identified in mice that commencement of doxycycline at seven days post vaccination (for *Brucella melitensis*) resulted in complete reduction of IgG specific antibodies for greater than 50 days. Conversely, if antibiotics were commenced 28 days post vaccination, IgG specific antibodies were observed to remain, although at lower levels, compared to no antibiotic treatment^32^. In the current koala trial, all animals had pre-existing *Chlamydia* infections that had persisted long enough to cause signs of cystitis. Although, the amount of time between infection and admission to the hospital was unknown, previous vaccine trials of healthy koalas indicate that systemic IgG antibodies take approximately 28 days to accumulate^2^. Together, this indicates that although koalas were vaccinated at the same time as antibiotic administration, the presence of an active antibody response to the current infection possibly limited the lasting effects doxycycline had on the vaccine induce immune response, allowing for the enhanced IgG recognition of the MOMP in vaccinated koalas observed.

In previous koala vaccine trials, significant effort has been employed to include multiple *C. pecorum* genotypes in an effort to induce a protective immune response to multiple circulating genotypes^26,27^. The rationale behind this development was due to the differences in circulating genotypes of *C. pecorum* within different populations^33^. However, in the current study, analysis of epitope mapping results from vaccinated koalas, it was determined that while variable MOMP domains are recognised by IgG antibodies, it was antibody production to conserved MOMP domains which were primarily enhanced during vaccination. These observations indicate that IgG antibodies primarily recognise the conserved domains of MOMP, in response to both natural infection and vaccination.

**Figure 6:**
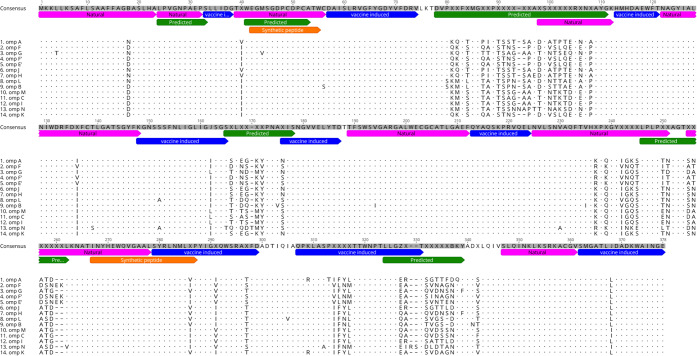
Predicted, natural and vaccine induced B-cell epitopes including previous synthetic peptide vaccine sequences aligned to 14 different *C. pecorum* MOMP sequences. Pink are sequences recognised during natural infection (current study), blue are recognised due to vaccination with recombinant MOMP protein (current study), green are in silico predicted B-cell epitopes^36^ and orange are peptide sequences used for vaccination^2^ (Performed using Geneious 9.1).

Computational analysis for B- and T-cell epitopes within the *Chlamydia* MOMP have been performed for many different *Chlamydia* species^34,35^ including *C. pecorum*^36^. These predictions identified B-cell and T-cell epitopes within both conserved and variable domains of the MOMP. However, when natural and vaccine induced IgG recognised epitopes are compared to these predicted sequences, discrepancies are observed (Fig. 6). Four of the predicted B-cell epitopes from Kollipara *et al*. (2013) align with naturally recognised epitopes, with the vaccine induced epitopes falling outside theses predicted regions, with exception of half a single predicted sequence aligning with a vaccine recognised sequence. Because the recombinant protein used in the vaccine is not in its natural conformation, it is reasonable that new regions would be recognised post vaccination.

Significant progress in the further development of the koala *Chlamydia* MOMP vaccine has resulted in the first published data of a synthetic peptide-based vaccine against *C. pecorum* in koalas. Nyari *et al*. (2018) details a vaccination regime using two synthetic peptides representing conserved regions of the *C. pecorum* MOMP (Fig. 6), compared to koalas vaccinated with the recombinant MOMP protein form of the vaccine^2^. Their results indicated that replacement of the recombinant full-length protein with smaller, synthetic peptides did induce an IgG and IgA antibody response similar to the rMOMP vaccine^2^. Their study indicated the feasibility of a synthetic peptide vaccine towards *C. pecorum* in koalas and suggested that a broader antigenic load may be required to increase the vaccine effectiveness. Results from our current work identified vaccine-specific B-cell epitopes recognised by circulating IgG antibodies. These vaccine-specific epitopes could be utilised in a synthetic peptide vaccine with the aim of increasing the antibody responses observed in the previous peptide vaccine trial. Furthermore, the selection of peptides recognised due to vaccination only and not natural infection should also be accounted for. With the majority (up to 80%) of successfully treated koalas returning with new infections, it is deemed important to induce an immune response that differs to natural responses.

In addition to B-cell antibody responses, transcriptomic profiling of PBMCs revealed an up-regulation of genes involved in neutrophil degranulation, within the first month post-vaccination. Until recently, neutrophils were predominately associated with innate immune responses, inflammation and disease^37^. However, recent studies have identified an increasingly diverse role for circulating neutrophils and their involvement in adaptive immune responses^37^. Studies performed in ovalbumin-induced immune responses in murine models have observed neutrophils acting as antigen presenting cells to CD8 + T-cells via major histocompatibility complex I mechanisms^38^. Studies of neutrophil-*Chlamydia* interactions have also identified that *Chlamydia* plasmids can attract and prolong neutrophil survival in the absence of TLR4 activation *in vitro*^39^, however the mechanisms for these responses are currently unknown. Further studies reported that the development of ocular pathology and the activation of an adaptive immune response in *C. caviae* ocular infections of guinea pigs, is neutrophil-dependent^40^. Finally, interactions between neutrophils and B-cells, within the marginal zone of the spleen, have been observed to have a positive effect on antibody production through the production of B-cell activation factors (BAFF), proliferation-inducing ligands (APRIL), and interleukin (IL)-21^41^. This study identified neutrophil degranulation within one month of vaccination through transcriptomic profiling. Collectively, our understanding of the role that neutrophils play in the adaptive immune response is continuing to evolve. The possible indication of adaptive immune responses involving neutrophils in this report, requires more detailed specific studies to uncover the mechanisms involved in B-cell to neutrophil interactions involved in adaptive immunity in the koala.

The adjuvant component to the vaccine has previously been shown to alter specific immune response pathways. Specifically, the carrier molecule PCEP has been demonstrated as a potent stimulant of IgG antibodies, with a threefold increase in titres when compared to aluminium hydroxide in mice^42^. Poly I:C combined with an anti-malaria vaccine was demonstrated to significantly increase antigen specific IgG antibodies compared to vaccination without Poly I:C^43^. Finally, IDR-1002 has been demonstrated *in vitro* to regulate neutrophil activity to promote adaptive immune functions and limit inflammation^44^.

Limitations to this trial were the use of chloramphenicol in koala K3. As many reports have demonstrated that different antibiotics can have differing effects on antibody responses, the use of two different antibiotics was not ideal. However, as the IgG antibody profiles from all koalas followed similar patterns, it could be assumed that both chloramphenicol and doxycycline have similar immunomodulatory effects, although this was in a limited number of koalas and needs further assessment. Further limitations include the outbred nature of the koalas and the small number of animals. This limitation resulted in limited understanding of the cellular immune responses observed through RNA sequencing techniques, however indications of neutrophil involvement allows for future detailed analysis of possible pathways involved in this regulation.

In conclusion, the present study demonstrates that antibiotic treatment has a temporary negative effect on circulating anti-MOMP IgG antibodies. Observations of koalas vaccinated with current signs of *Chlamydia*-induced cystitis indicated that after the antibiotic treatment had been completed, the systemic anti-MOMP IgG antibody response increased in recognition and intensity to both conserved and variable domains of the *C. pecorum* MOMP. Furthermore, analysis of genetic transcription within PBMCs indicated an increase in neutrophil degranulation. Finally, IgG recognition of specific amino acids within conserved domains of the MOMP indicated that the representation of multiple MOMP genotypes within future vaccine studies may not be necessary and these identified sequences should be utilised to increase immune responses using synthetic peptides as antigenic targets. Collectively, this study has increased our understanding of vaccination during antibiotic treatment and created new avenues for continued improvement in koala health management through vaccination. Finally, these findings indicate that the use of antibiotics during vaccination do not affect antigenic recognition of the vaccine.

### Animal ethics approvals

Ethical approval for this study was granted by University of the Sunshine Coast, Animal Ethics Committee (AEC CA No. AN/S/17/49) and was performed under a Queensland Scientific Research Purposes Permit granted by Queensland Government, Department of Environment and Heritage Protection (SPP No. WA0001189).

## Materials and methods

### Animal trial

Ten koalas, five male and five female (between three and five years of age), were included in this trial. These koalas presented to Currumbin Wildlife Hospital (CWH) or Australia Zoo Wildlife Hospital (AZWH) with signs of cystitis and were assessed using ultrasound and visual inspection. All koalas from CWH were transported to AZWH for treatment and re-examined on arrival to confirm the diagnosis of cystitis. Upon diagnosis, each koala was administered antibiotics (Doxycycline, 5 mg/kg diluted 50:50 in sterile saline, given subcutaneously, once per week for four weeks or chloramphenicol, 60 mg/kg given subcutaneously once per day for 28 days) and admitted to the hospital for eight weeks, as per standard hospital procedures.

### Sampling procedures

Prior to the commencement of antibiotic treatment and every two weeks for the following eight weeks, each koala had urogenital, rectal and ocular swabs and whole blood (3–5 mL) collected in an EDTA collection tube (Interpath Services). Urogenital (urethral for males and urogenital sinus for females), conjunctival and rectal swabs collected from koalas at each time point were swirled in 500 µL of sterile PBS and stored at −20 °C. The whole blood sample was transported to the laboratory within 2 hours where 1 mL was separated for plasma (centrifuged at 133100 RPM for 10 min) and the remaining 2–4 mL was fractioned for peripheral blood mononuclear cells (PBMC) using Ficoll within a 15 mL Sepmate tube (STEMCELL Technologies), as per manufacturer instructions. The PBMC fraction was stored in 200 µL of RNAlater (Invitrogen) at −80 °C.

All examinations were performed under anaesthesia of an intramuscular (quadriceps muscle group) injection of 3 mg/kg alfaxalone (Alfaxan CD RTU, Jurox). Once the animal was sedated, it was fully anaesthetised with 4–5% isoflurane gas and 1.5–2 L of oxygen delivered via a face mask. Sedation was maintained with 1.5–2% isoflurane gas and 1.5–2 L of oxygen for the duration of the examination^45^.

### Vaccine preparation and administration

The vaccine antigens used were three recombinant proteins representing the *C. pecorum* MOMP genotypes A, F and G^26,33,36^. These MOMP genotypes were amplified and sequenced from known *C. pecorum* strains isolated from South East Queensland koalas. Amplicons were cloned into the pRSET-A bacterial plasmid (Invitrogen) and transformed into *E. coli* BL21 cells, as previously described^27^. For expression in the current project, each *E. coli* strain was grown at 37 °C for approximately 3 hours (to achieve a 600 nm optical density between 0.2 and 0.5) before 1 mM of isopropyl β-D-1-thiogalactopyranoside (IPTG) was added to activate the T7 promoter of the pRSET-A plasmid. Cultures were further incubated at 27 °C for 12 hours before recombinant protein was isolated, as previously described with minor modification^27^. The subsequent rMOMP proteins were then isolated utilising the histidine tag on TALON affinity beads in an 8 M Urea buffer, resuspended in PBS using snake skin dialysis tubing and concentrated using a 30 kDa cut off protein concentrator^27^. The final concentrated protein elution was then quantified using a BSA protein assay (Thermo Scientific), assessed for *E. coli* endotoxin concentration using the LAL chromogenic endotoxin quantification kit (Pierce) and visualised on SDS-PAGE (Supplementary Figure 2, as previously described^27^.

The vaccine antigen containing rMOMPs A, F and G (50 μg each rMOMP protein) was combined with a three-component adjuvant, containing Poly I:C (250 μg), Host Defence Peptide-Innate Defence Regulator IDR-1002 (500 μg), and Polyphosphazene EP3 (250 μg) (VIDO-Intervac, University of Saskatchewan, Canada), to a total vaccine volume of 500 μL with sterile endotoxin-free PBS. Each vaccine was prepared in a sterile endotoxin-free amber glass vial, stored on ice and administered within 5 hours of preparation. Post clinical examination and sample collection, five koalas received a single subcutaneous vaccine injection during admission to AZWH (three male and two female)^27^. Unfortunately, four koalas died before the end of the trial (two unvaccinated, a male at week 6 and a female at week 2, two vaccinated, a male at week 2 and a female at week 4), from either antibiotic treatment failure or gastrointestinal dysbiosis. Vaccination did not cause any adverse health outcome in this trial.

### PBMC sample processing

Total RNA was extracted from all PBMC 200 µL fractions using the Qiagen, RNeasy Mini Kit (Venlo, The Netherlands) following the “RNA Purification from animal cells, Spin Protocol”. All isolated RNA was further treated with TURBO DNA-free (Life technologies, Carlsbad, USA) as per manufacturer instructions. Finally all isolated RNA was precipitated in ethanol, to remove any contaminating salts, as previously described^46^.

### Epitope mapping of koala IgG plasma to *C. pecorum* MOMP

As previously described, library of 88 biotinylated 15 amino acid peptides was used to screen the full-length koala *Chlamydia pecorum* MOMP sequence for antibodies to MOMP F, four variable domains of *C. pecorum* MOMP A, G and H and three peptides representing the MOMP conserved leader sequence, cleaved post protein translation^26^. The detection of epitope-specific koala IgG was performed as previously described with minor changes^26^. The wells of bovine serum albumin blocked, 96-well streptavidin coated plates (Thermo Scientific) were coated individually with each of the 88 biotinylated peptides at a concentration of 2 µg/well in 1X PBS tween-20 (0.01%) and incubated at room temperature for one hour on a plate shaker. Plates were then washed four times using a Bio-Rad plate washer with 200 µL per well with PBS-Tween-20 (0.05%). Post washing, the individual plasma samples from weeks 0, 4 and 8 from six koalas (three vaccinated and three unvaccinated) were diluted 1:100 in PBS-tween-20 (0.01%) and 100 µL was added to the wells, incubated and washed as before. The plates were then incubated with sheep anti-koala IgG (1:8000 in PBS-tween-20 (0.01%)) (100 µL/well), incubated and washed as before. Plates were then incubated with HRP-labelled rabbit anti-sheep IgG (1:20,000 in PBS-tween-20 (0.01%)) (100 µL/well), incubated as previously and washed five times with 200 µL per well with PBS. Finally, each well was incubated with 100 µL/well of tetramethyl-benzidine dissolved in phosphate citrate buffer containing sodium perborate dimethyl sulfoxide (1:10) and 30% hydrogen peroxide (1:5000) at room temperature for 20 minutes, protected from light, after which 100 µL/well of 1 M sulphuric acid was added and the optical density measured at 450 nm (EnSpire, 96-well plate reader).

### Koala specific *C. pecorum* IgG and IgA ELISA for mucosal swabs

*C. pecorum* specific mucosal IgG and IgA recognition from urogenital, conjunctival and rectal swabs were determined using ELISA techniques to recombinant MOMP genotype G or heat inactivated semi-purified *C. pecorum* genotype G elementary bodies (EBs) (purified as per^47^) as previously described^23^.

### Total RNA sequencing

Total RNA sequencing was performed on both vaccinated (three male and two female) and unvaccinated (two male and two female) koala PBMC fractions collected at admission, week two, week four, week six and week eight. Isolated RNA (method described above) were sent to the University of New South Wales, Ramaciotti Centre for Genomics for library preparation and sequencing. Library preparation was performed using the SMARTer stranded total RNA-Seq kit v2 - Pico input mammalian-strand-specific preparation kit. Sequencing was performed with a NextSeq. 2 × 75 bp high output Illumina sequencer.

### Sequence quality control and mapping to the koala genome

Bioinformatics processing and analysis of all sequences was performed as previously described^46^ using the published koala annotated genome (GCF_002099425.1) and bioinformatics programs STAR (version 2.6), HTseq (version 0.11.2) and R (version 3.5.2). Sequence reads have been deposited to SRA, Bio Project identification number PRJNA540172.

### Statistical analysis methods

Antibody recognition analysis of the optical density readings (450 nm) for each sample were analysed using a Pearson’s correlation analysis and visualised in a heat map, constructed using the program pheatmap (version 1.0.12) on the platform R (version 3.3.1)^48^. Expression analysis of RNA sequencing read counts were analysed using the statistical program EdgeR (version 3.24.3) on the platform R (version 3.5.2)^48^, using a quasi-likelihood approach (glmQLFit). Differential gene expression was compared between unvaccinated and vaccinated groups during the first and second months post vaccination. Differentially expressed genes identified as significant between the vaccinated and unvaccinated groups were analysed using the on-line analysis tool “Reactome pathway knowledgebase” against known pathways present in *Homo sapiens*^49^.

## Supplementary information

Supplementary Information.
