# Supplementary material for: Vaccination of koalas during antibiotic treatment for Chlamydia-induced cystitis induces an improved antibody response to Chlamydia pecorum
Source: Sci Rep. 2020 Jun 23;10:10152. doi: 10.1038/s41598-020-67208-x (PMC7311432; doi:10.1038/s41598-020-67208-x)
Supplement: Supplementary file 1 — Supplementary Information. [file 41598_2020_67208_MOESM1_ESM.docx]

Vaccination of koalas during antibiotic treatment for *Chlamydia*-induced cystitis induces an improved antibody response to *Chlamydia pecorum*

Samuel Phillips^a^, Bonnie L Quigley^a^, Olusola Olagoke^a^, Rosemary Booth^b^, Michael Pyne^c^, Peter Timms^a*^

Genecology Research Centre, School of Science and Engineering, The University of the Sunshine Coast, Queensland, Australia ^a^; Australia Zoo Wildlife hospital, Steve Irwin Way, Australia ^b^; Currumbin Wildlife Hospital, Currumbin, Australia ^c^

*Corresponding author [ptimms@usc.edu.au](mailto:ptimms@usc.edu.au)


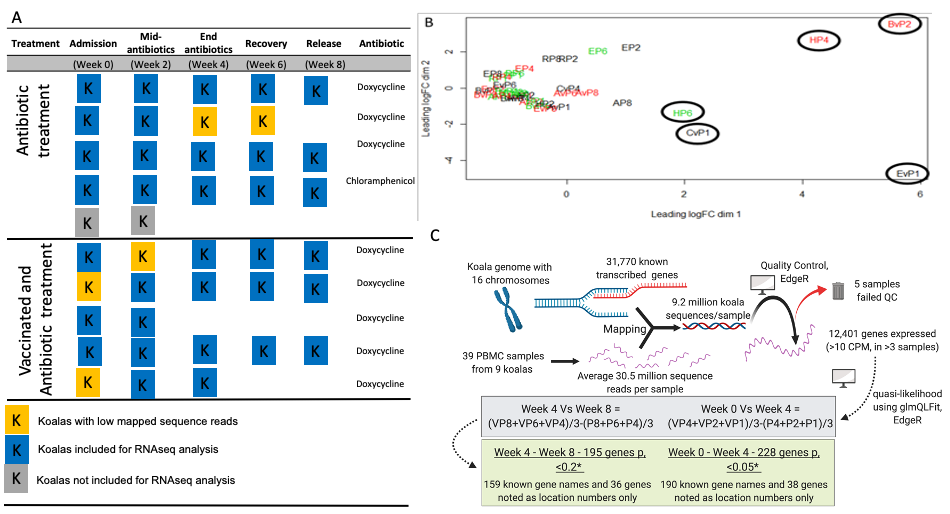


Supplementary figure 1. Bioinformatics approach to RNA sequencing analysis. A) Identification of koalas included in the final differential gene expression analysis, with orange coloured koalas circled in part B. B) Multidimensional scaling plot used to identify sample similarity. Samples circled in black were removed from analysis due to the decreased similarity to the majority of the sample population produced using the program EdgeR in R Studio. C) Bioinformatics analysis of sequences, including mapping to the koala reference genome, quality control (>10 counts per million (CPM) in >3 samples), quasi-likelihood statistical analysis (using glmQLFit) approach and filtering to differentially expressed genes (DEGs) (adjusted p < 0.05 and <0.2) (V = Vaccinated koalas, P = PBMCs) drawn using the online program Bio render.

* False Discovery Rate (FDR) adjusted p value

**A**

1 2 3 4 5 6 7

| **Lane** | **Sample** |
| --- | --- |
| 1 | Flow through |
| 2 | Flow through |
| 3 | Wash 1 |
| 4 | Wash 2 |
| 5 | Imidazole (10mM) Wash |
| 6 | Elution - Imidazole (200mM) |
| 7 | Protein Ladder (Bio-Rad) |


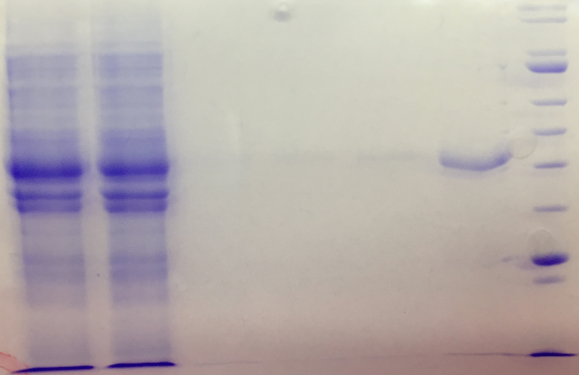


**B**

1 2 3 4 5 6 7


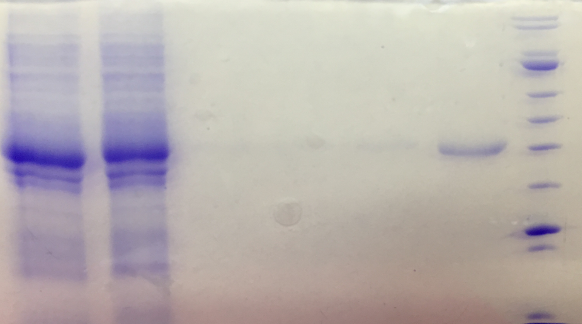


**C**

1 2 3 4 5 6 7


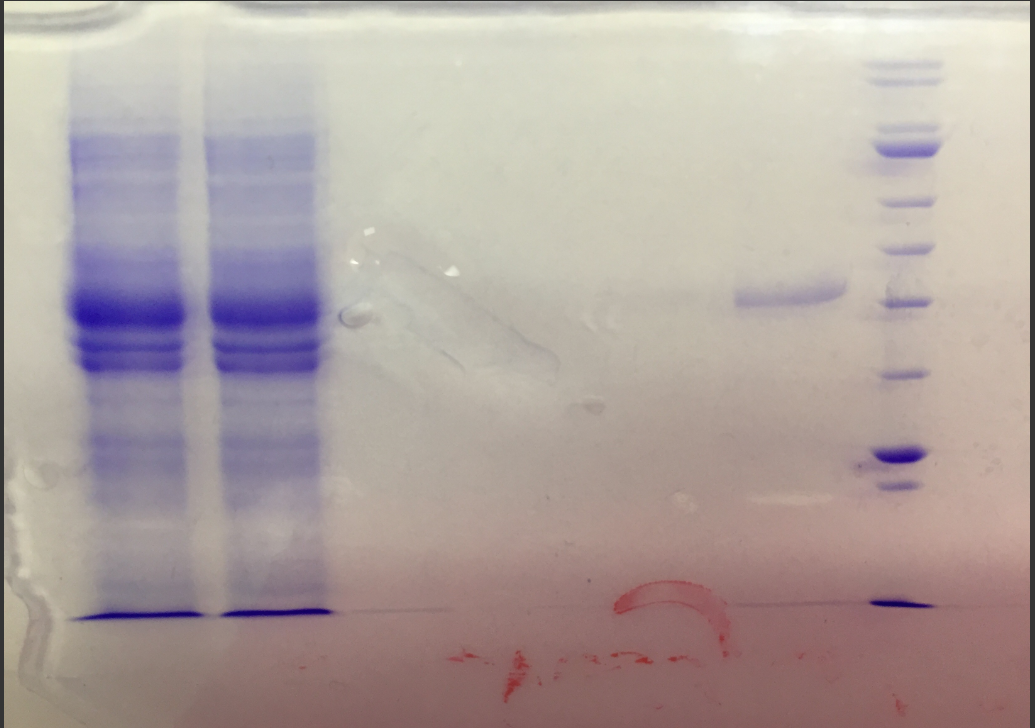
 Supplementary figure 2. SDS PAGE visualisation of purified MOMP recombinant proteins with Bio-Rad protein ladder. Visualised using 0.75 mm wide 5% on 12% double layer SDS-PAGE gel (110 V for 1 h) stained with Coomassie blue gel stain, arrows indicate 40kda protein marker. A) Concentrated and purified MOMP F recombinant proteins, B) Concentrated and purified MOMP G recombinant proteins, C) Concentrated and purified MOMP A recombinant proteins
